# Elevated remnant cholesterol is linked to non-alcoholic fatty liver disease in patients with type 2 diabetes mellitus

**DOI:** 10.3389/fnut.2026.1782646

**Published:** 2026-04-16

**Authors:** Hang Zhao, Zhimei Zhang, Yunjia Zhang, Wenhui Jiang, Cuijuan Qi, Luping Ren, Shuchun Chen

**Affiliations:** 1 Department of Endocrinology, Hebei General Hospital, Shijiazhuang, Hebei, China; 2 Geriatrics, Harbin 242 Hospital, Harbin, Heilongjiang, China

**Keywords:** lipid, non-alcoholic fatty liver disease, remnant cholesterol, restricted cubic spline, type 2 diabetes mellitus

## Abstract

**Objective:**

The aim of this study was to investigate the association between remnant cholesterol (RC) levels and non-alcoholic fatty liver disease (NAFLD) in patients diagnosed with type 2 diabetes mellitus (T2DM).

**Participants and methods:**

This was a cross-sectional study involving 308 patients with T2DM who were hospitalized at Hebei General Hospital. The participants were categorized into a NAFLD group and a non-NAFLD (control) group, and comparative analyses were conducted between the groups. Regression analysis, subgroup analysis, and interaction tests were performed. Additionally, restricted cubic spline (RCS), predicted probability, and sensitivity analyses were used to explore and verify the association between RC levels and NAFLD.

**Results:**

Elevated RC levels were associated with NAFLD (OR = 4.23, 95% CI: 1.73–12.04) after adjusting for all confounding factors, regardless of whether RC was treated as a continuous or tertile variable. No significant interactions were observed for any of the subgroups, including age (*P* = 0.772), gender (*P* = 0.231), diabetes duration (*P* = 0.757), BMI (*P* = 0.221), and HbA1c (*P* = 0.916). RCS analysis indicated that there was a significant association between RC levels and NAFLD (*P* overall = 0.006), and the relationship was linear (*P* nonlinear = 0.399). The mean predicted probability of NAFLD increased with increasing RC concentration, rising from 65.7% (95% CI: 53.6%−76.1%) at 0.2 mmol/L to 85.8% (95% CI: 76.9%−91.7%) at 1.0 mmol/L. In the sensitivity analysis, when RC was categorized into tertiles, the association between RC levels and NAFLD remained significant across all models (Model 3: Tertile 3 vs. Tertile 1, OR = 2.05, 95% CI: 1.06–4.03, *P* = 0.035). Furthermore, a significant linear trend was observed (*P* trend = 0.035), confirming the robustness of the primary findings.

**Conclusion:**

In patients with T2DM, RC levels are independently associated with NAFLD.

## 1 Introduction

Data from the International Diabetes Federation indicate that approximately 10.5% of the global population is affected by diabetes, a figure projected to rise to 12.5% by 2045 (1). Among these cases, type 2 diabetes mellitus (T2DM) accounts for the largest proportion. Furthermore, non-alcoholic fatty liver disease (NAFLD) poses a growing public health challenge worldwide, with a global prevalence estimated to be as high as 30.05% (2). In China, the prevalence reaches 29.6%—specifically, 34.8% in males and 23.5% in females—representing a substantial disease burden (3). Both T2DM and NAFLD are classified as metabolic diseases (4). NAFLD is characterized as a chronic, progressive liver condition that affects genetically predisposed individuals, primarily resulting from over nutrition and insulin resistance. The spectrum of NAFLD encompasses non-alcoholic fatty liver and non-alcoholic steatohepatitis (NASH), alongside associated fibrosis or cirrhosis (5). NAFLD, metabolic syndrome, and T2DM are interrelated and collectively contribute to the development of atherosclerotic cardiovascular disease, chronic kidney disease, liver decompensation, and hepatocellular carcinoma, thereby posing an escalating public health challenge (6, 7).

Both NAFLD and T2DM are intricately associated with dyslipidemia. Besides conventional lipid metrics, recent studies have explored the utility of novel parameters such as remnant cholesterol (RC). RC refers to the cholesterol present in triglyceride-rich lipoproteins (TRLs), which include very low-density lipoprotein (VLDL), intermediate-density lipoprotein (IDL), and chylomicron remnants (8).

Evidence suggests that elevated RC levels are not only significantly associated with an increased risk of cardiovascular diseases but also contribute to the pathogenesis and progression of NAFLD. However, the specific relationship between RC and NAFLD in patients with T2DM remains inadequately characterized. The purpose of this study was to explore the association between RC levels and NAFLD in patients with T2DM, aiming to provide novel insights for the clinical prevention and management of NAFLD in this high-risk population.

## 2 Participants and methods

### 2.1 Study design and population

This was a cross-sectional study involving patients with T2DM admitted to the Department of Endocrinology at Hebei General Hospital during the period between 2023 and 2025. The study was approved by the Institutional Ethics Committee (20230128). All participants provided written informed consent.

### 2.2 Inclusion and exclusion criteria

The inclusion criteria were ≥18 years and a clinical diagnosis of T2DM as defined by the World Health Organization.

The exclusion criteria were: (1) individuals diagnosed with alcoholic fatty liver; (2) those with acute diabetic complications, such as diabetic ketoacidosis or hyperglycemic hyperosmolar state; (3) patients with a history of fractures, surgical interventions, severe cardiovascular or cerebrovascular events within the preceding 3 months; and (4) individuals with lung or urinary tract infections within the preceding 3 months.

### 2.3 Diagnosis of NAFLD

The diagnosis of NAFLD involved the identification of hepatic steatosis based on ultrasound imaging. Identification was conditional on the exclusion of excessive alcohol consumption (defined as >30 g/day for men and >20 g/day for women) or the presence of other liver diseases, including viral hepatitis, toxic liver injury, alcoholic liver disease, and autoimmune hepatitis, among others.

### 2.4 Data collection

Two researchers collected and verified the information and data. The data included: (1) general characteristics, such as gender, age, body mass index (BMI), diabetes course, family history of diabetes, smoking history, and hypertension history; (2) glycated hemoglobin (HbA1c); (3) lipid profiles, including total cholesterol (TC), triglyceride (TG), high-density lipoprotein cholesterol (HDL-C), and low-density lipoprotein cholesterol (LDL-C); and (4) other indicators, such as total protein, albumin, uric acid (UA), blood urea nitrogen (BUN), creatinine (Cr), and 25-hydroxyvitamin D (25OHD). All patients fasted for 6–8 h overnight, and venous blood samples were collected the following morning for laboratory measurements. Biochemical parameters were measured using a fully automated biochemical analyzer (Canon PBA-FX8). HbA1c was measured by high-performance liquid chromatography using an HbA1c analyzer (HA-8190V). LDL-C was directly measured, and 25OHD levels were determined using a chemiluminescence immunoassay analyzer (MAJLUMI4000 plus).

### 2.5 Calculation of RC contents

RC (mmol/L) = TC – LDL-C – HDL-C.

### 2.6 Statistical analysis

Statistical analyses were performed using SPSS 21.0 (IBM Corp., Armonk, NY, USA) and R 4.5.1 (R Foundation for Statistical Computing, Vienna, Austria). A two-tailed *P*-value of < 0.05 was considered statistically significant. Categorical variables are reported as event counts and percentages, while continuous variables are expressed as means ± standard deviation for normally distributed data, or as median [25th percentile (P25), 75th percentile (P75)] for severely skewed data. Comparisons between groups were conducted using the chi-square test for categorical variables and either *t*-tests or non-parametric tests for continuous variables. Candidate variables for multivariable logistic regression were identified based on statistical significance in between-group comparisons (*P* < 0.05). To ensure model stability and avoid overfitting given the sample size (*n* = 308, including 211 NAFLD events), a conservative approach to covariate selection was adopted. Subsequently, multicollinearity was evaluated using the generalized variance inflation factor (GVIF). For categorical variables, the adjusted GVIF [GVIF^1/(2 × *Df*)^] was used to ensure comparability with continuous variables. A threshold of >5 was applied to both continuous and categorical variables. Additionally, a correlation coefficient |*r*|>0.5 among continuous variables was considered indicative of collinearity. Owing to collinearity between TG and RC, TG was excluded from the regression model. As RC is calculated as TC – LDL-C – HDL-C, these three components were also excluded. None of the variables ultimately included in the model showed collinearity. Binary logistic regression analysis was performed to explore the association between RC and NAFLD, with results presented as odds ratios (OR) and 95% confidence intervals (CIs). Three models were constructed: Model 1 was unadjusted; Model 2 was adjusted for age and diabetes course; and Model 3 was further adjusted for BMI, UA, and albumin. Model performance was evaluated using two approaches. Calibration was assessed using the Hosmer-Lemeshow goodness-of-fit test, where a *P*-value of >0.05 indicates an adequate fit. Discrimination was quantified by the area under the receiver operating characteristic curve (AUC), with corresponding 95% CIs calculated using the DeLong method. An AUC of >0.7 was considered to represent acceptable discrimination.

To examine whether the association between RC and NAFLD varied across populations, interaction terms (RC × subgroup variable) were introduced into the fully adjusted model (Model 3). Interaction *P*-values were calculated for each subgroup, including age (< 60 years vs. ≥60 years), sex (male *vs*. female), diabetes course (< 10 years vs. ≥10 years), BMI (non-obesity *vs*. obesity), and HbA1c (< 9% vs. ≥9%). An interaction effect was defined by a *P*-value for interaction of < 0.05. Stratified subgroup analyses were performed within each category using the same adjustments as those in Model 3. A restricted cubic spline (RCS) was used to explore the dose-response relationship between RC and NAFLD, with the determination of both overall *P* and non-linear *P*. To select the optimal number of knots for the RCS analysis, models with 3, 4, and 5 knots were compared using the Akaike Information Criterion (AIC). As the 3-knot model yielded the smallest AIC value, this model was selected. The three knots were placed at the 25th, 50th, and 75th percentiles of the RC distribution, using the median RC value (0.5 mmol/L) as the reference. This RCS model incorporated the same covariates as Model 3 (age, BMI, UA, albumin, and diabetes duration). Both the overall association (*P* for overall) and the non-linear component (*P* for nonlinear) were reported. The RCS plot was augmented with a histogram showing the frequency distribution of RC values to visualize the data density across the RC range. To enhance clinical interpretability, marginal predicted probabilities of NAFLD were calculated across the spectrum of RC values based on the fully adjusted model (Model 3). For each RC level, the predicted probability was obtained by averaging individual predictions over the covariate distribution of the study population. To assess the robustness of the findings, a sensitivity analysis was performed by categorizing RC into tertiles based on the 33.3rd and 66.7th percentiles of its distribution. Tertile 1 served as the reference group. Logistic regression was repeated using the tertile groups in two ways: as a categorical variable to estimate ORs for Tertile 2 and Tertile 3 compared to Tertile 1, and as an ordinal variable to test for a linear trend (*P* trend) across increasing RC tertiles. This analysis was conducted for all three models. To evaluate whether the sample size was sufficient to detect the observed association between RC and NAFLD, a *post-hoc* power calculation was performed. Based on a total sample size of 308 participants, a NAFLD prevalence of 69%, an observed OR of 4.23 per 1 mmol/L increase in RC (derived from the fully adjusted model), and a two-sided significance level of 0.05, the study had 99.4% power to detect this effect. This indicated that the primary analysis had adequate power.

## 3 Results

### 3.1 General characteristics

This study included 308 patients diagnosed with T2DM, with a mean age of 55.7 ± 12.0 years. Among the participants, 202 were male, accounting for 65.6% of the cohort. The median RC was measured at 0.5 (0.3, 0.8) mmol/L, and 211 patients in this population had NAFLD, representing a prevalence of 69.0%.

### 3.2 Group comparisons

The study participants were categorized into a control (non-NAFLD) group and a NAFLD group. Compared to controls, patients with NAFLD were younger, had a higher BMI, and exhibited elevated TG, RC, UA, and albumin levels. Conversely, the levels of HDL-C and the proportion of patients with a diabetes duration of < 10 years were significantly lower (*P* < 0.05) in the NAFLD group (Table 1).

**Table 1:** Clinical characteristics of patients with T2DM by NAFLD.

Characteristic	Overall	Non-NAFLD	NAFLD	*P* value
	**(*****N*** = **308, 100%)**	**(*****N*** = **97, 31%)**	**(*****N*** = **211, 69%)**	
Age (years)	55.7 ± 12.0	58.7 ± 11.3	54.3 ± 12.1	0.002^*^
Age group (*n*, %)				
< 60 years	185 (60.1%)	51 (52.6%)	134 (63.5%)	0.090
≥60 years	123 (39.9%)	46 (47.4%)	77 (36.5%)	
Gender group (*n*, %)				
Female	106 (34.4%)	34 (35.1%)	72 (34.1%)	0.976
Male	202 (65.6%)	63 (64.9%)	139 (65.9%)	
Smoking history (*n*, %)	96 (31.2%)	26 (26.8%)	70 (33.2%)	0.323
Drinking history (*n*, %)	77 (25.0%)	18 (18.6%)	59 (28.0%)	0.103
Family history (*n*, %)	111 (36.0%)	27 (27.8%)	84 (39.8%)	0.057
Hypertension history (*n*, %)	135 (43.8%)	40 (41.2%)	95 (45.0%)	0.618
BMI (kg/m^2^)	25.8 ± 3.4	23.9 ± 3.2	26.7 ± 3.1	< 0.001^*^
BMI group (*n*, %)				
No obesity	232 (75.3%)	85 (87.6%)	147 (69.7%)	0.001^*^
Obesity	76 (24.7%)	12 (12.4%)	64 (30.3%)	
Diabetes course (*n*, %)				
< 10 years	177 (57.5%)	47 (48.5%)	130 (61.6%)	0.041^*^
≥10 years	131 (42.5%)	50 (51.5%)	81 (38.4%)	
HbA1c (%)	8.7 (7.3, 10.4)	8.4 (6.9, 10.4)	8.8 (7.5, 10.5)	0.155
HbA1c group (*n*, %)				
< 9%	168 (54.5%)	56 (57.7%)	112 (53.1%)	0.523
≥9%	140 (45.5%)	41 (42.3%)	99 (46.9%)	
TC (mmol/L)	4.7 ± 1.1	4.5 ± 1.1	4.8 ± 1.1	0.148
TG (mmol/L)	1.5 (1.1, 2.2)	1.1 (0.8, 1.6)	1.7 (1.2, 2.4)	< 0.001^*^
HDL (mmol/L)	1.0 ± 0.2	1.1 ± 0.2	1.0 ± 0.2	< 0.001^*^
LDL (mmol/L)	3.1 ± 0.8	2.9 ± 0.9	3.2 ± 0.8	0.071
RC (mmol/L)	0.5 (0.3, 0.8)	0.4 (0.3, 0.6)	0.6 (0.4, 0.8)	< 0.001^*^
UA (μmol/L)	309.1 ± 83.1	294.7 ± 71.8	315.8 ± 87.2	0.032^*^
Total protein (g/L)	67.6 ± 6.8	66.6 ± 8.6	68.1 ± 5.8	0.120
Albumin (g/L)	41.3 ± 3.2	40.8 ± 3.1	41.5 ± 3.2	0.044^*^
Cr (μmmol/L)	71.6 (62.6, 80.5)	72.9 (63.1, 82.6)	70.5 (62.5, 78.7)	0.204
BUN (mmol/L)	5.1 (4.2, 6.1)	5.3 (4.4, 6.3)	5.0 (4.1, 6.0)	0.132
25OHD (ng/mL)	18.2 (13.8, 22.2)	19.2 (14.2, 23.6)	17.9 (13.8, 21.3)	0.154
DN (*n*, %)	49 (15.9%)	15 (15.5%)	34 (16.1%)	>0.999
DN (*n*, %)	86 (27.9%)	30 (30.9%)	56 (26.5%)	0.509
DPN (*n*, %)	221 (71.8%)	68 (70.1%)	153 (72.5%)	0.764

Data were expressed as number (%) or mean ± SD/median (P25, P75). ^*^P < 0.05 indicates statistical significance. ApoA1, apolipoprotein A1; ApoB, apolipoprotein B; BMI, body mass index; BUN, blood urea nitrogen; Cr, creatinine; DM, diabetes mellitus; DN, diabetic nephropathy; DPN, diabetic peripheral neuropathy; DR, diabetic retinopathy; FBG, fasting blood glucose; HbA1c, glycated hemoglobin; HDL-C, high-density lipoprotein cholesterol; LDL-C, low-density lipoprotein cholesterol; NAFLD, non-alcoholic fatty liver disease; RC, remnant cholesterol; SD, standard deviation; T2DM, type 2 diabetes mellitus; TC, total cholesterol; TG, triglyceride; 25OHD, 25-hydroxyvitamin D.

### 3.3 Association between RC and NAFLD

In Model 1, which was unadjusted for confounding factors, RC was positively associated with NAFLD (OR = 6.49, 95% CI: 2.56–18.16). After adjusting for age and diabetes course in Model 2, a positive relationship remained (OR = 5.35, 95% CI: 2.10–15.06). In the fully adjusted Model 3, which incorporated BMI, UA, and albumin, each 1 mmol/L increase in RC was associated with a 4.23-fold increase in the odds of NAFLD (OR = 4.23, 95% CI: 1.73–12.04) (Table 2). Model 3 demonstrated good calibration (Hosmer-Lemeshow test: χ^2^ = 4.56, *df* = 8, *P* = 0.803) and acceptable discrimination, with an area under the curve (AUC) of 0.778 (95% CI: 0.723–0.834).

**Table 2:** Regression analysis of RC and NAFLD with RC as a continuous variable.

Models	NAFLD events/total population	OR	95% CI	*P* value
Model 1	211/308	6.49	2.56, 18.16	< 0.001^*^
Model 2	211/308	5.35	2.10, 15.06	< 0.001^*^
Model 3	211/308	4.23	1.73, 12.04	0.004^*^

Data were expressed as OR (95% CI). ^*^P < 0.05 indicates statistical significance.

Model 1: crude model.

Model 2: adjusted for age and diabetes course.

Model 3: adjusted for age, diabetes course, BMI, UA, albumin. BMI, body mass index; CI, confidence interval; NAFLD, non-alcoholic fatty liver disease; OR, odds ratio; RC, remnant cholesterol; UA, uric acid.

### 3.4 Subgroup analysis

**Figure 1:**
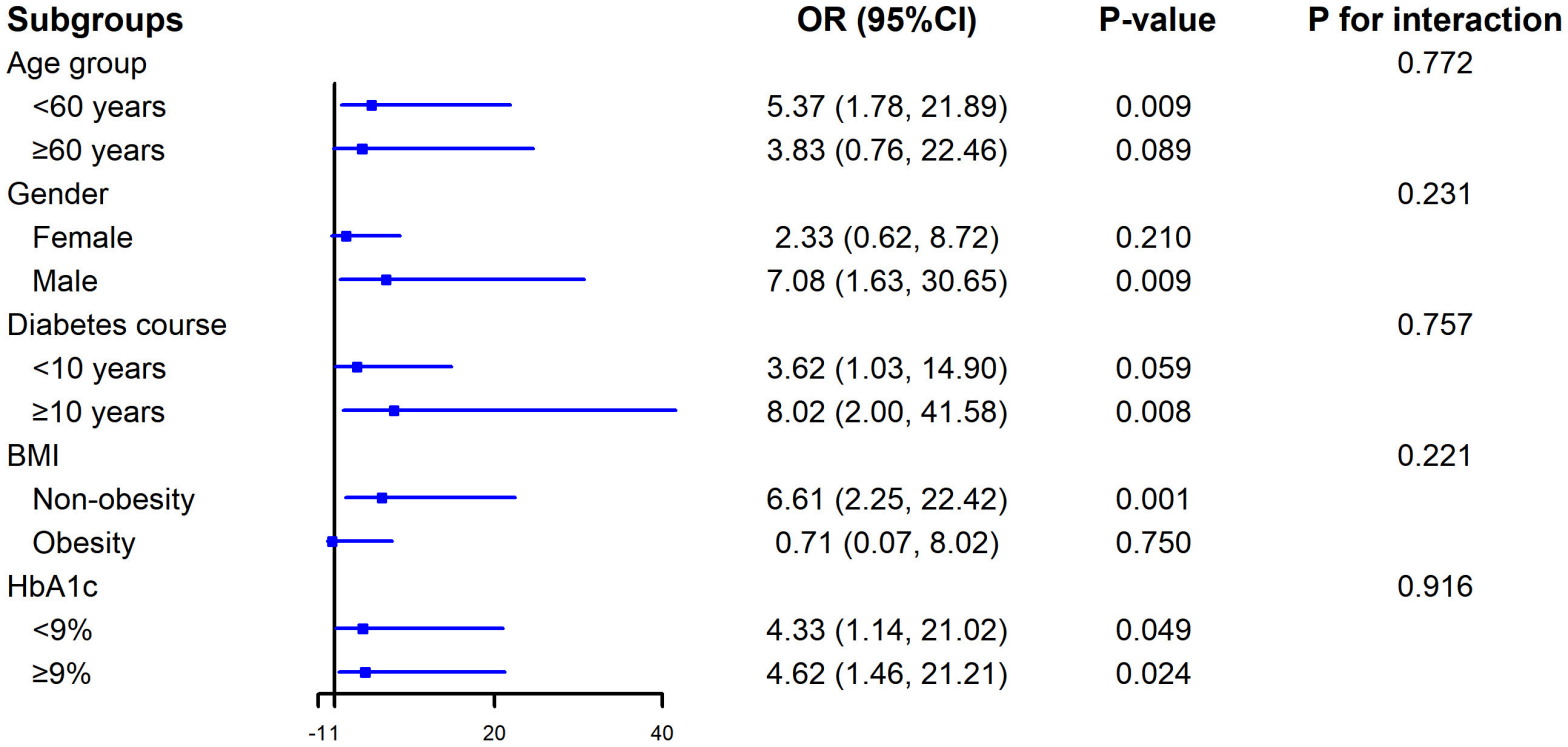
Subgroup analysis of the association between RC and NAFLD. BMI, body mass index; HbA1c, glycated haemoglobin; NAFLD, non-alcoholic fatty liver disease; RC, remnant cholesterol.

The results of the subgroup analysis showed that RC was positively associated with NAFLD among individuals aged < 60 years (OR = 5.37, 95% CI: 1.78–21.89), males (OR = 7.08, 95% CI: 1.63–30.65), those with a diabetes course ≥10 years (OR = 8.02, 95% CI: 2.00–41.58), and non-obese participants (OR = 6.61, 95% CI: 2.25–22.42). However, interaction tests revealed no significant interactions for any of the subgroups, including age (*P* = 0.772), gender (*P* = 0.231), diabetes course (*P* = 0.757), BMI (*P* = 0.221), and HbA1c (*P* = 0.916) (Figure 1). These results suggested that the association between RC and NAFLD remained consistent across these subgroups.

### 3.5 Dose-response relationship between RC and NAFLD

**Figure 2:**
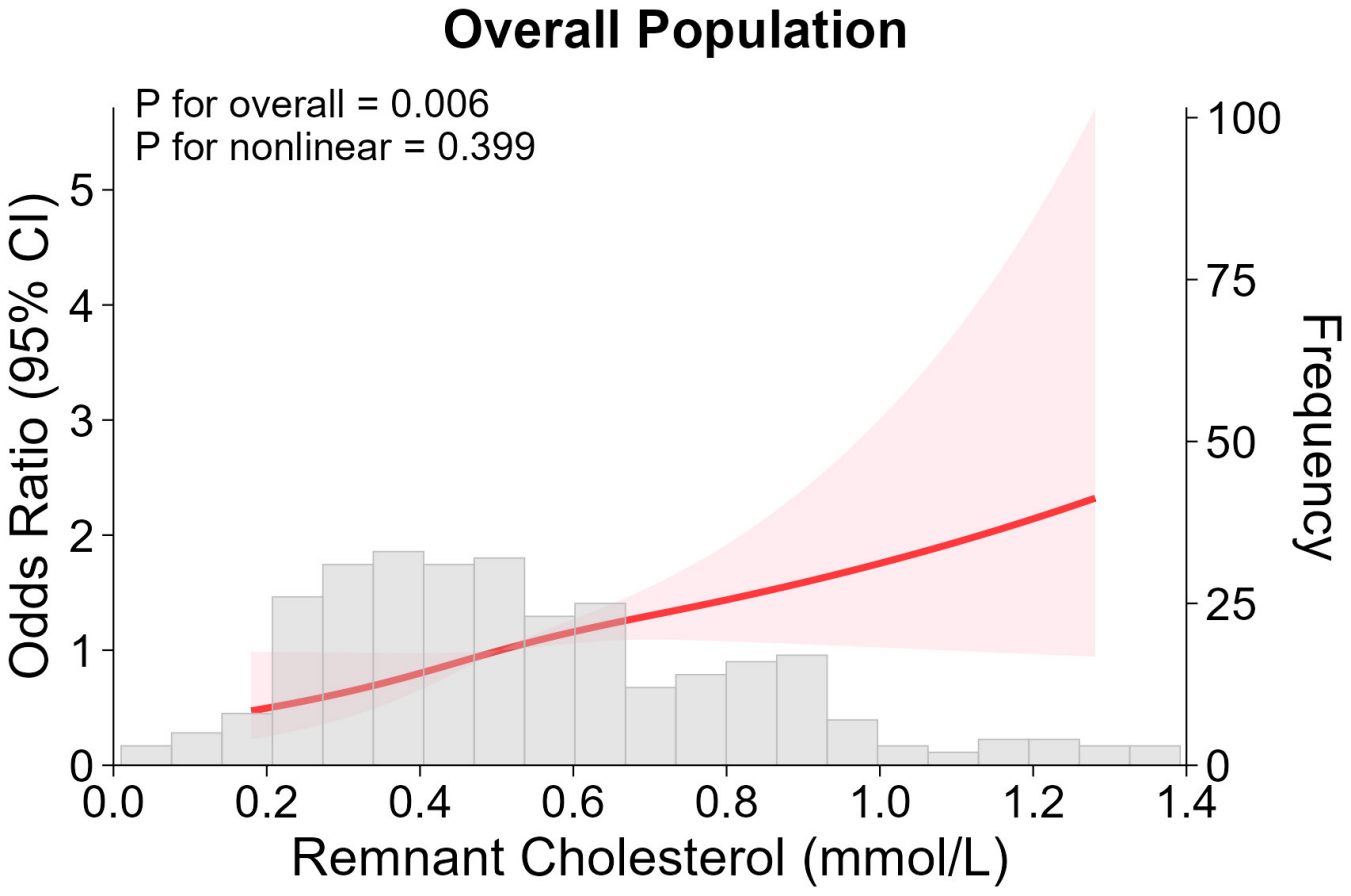
Restricted Cubic Spline analysis of RC and NAFLD. NAFLD, non-alcoholic fatty liver disease; RC, remnant cholesterol.

RCS analysis demonstrated that there was a significant association between RC and NAFLD (*P* overall = 0.006), with no evidence of a non-linear relationship (*P* nonlinear = 0.399). These results showed that the risk for NAFLD rises gradually with increasing RC concentrations (Figure 2).

### 3.6 Predicted probability of NAFLD based on RC concentrations

**Figure 3:**
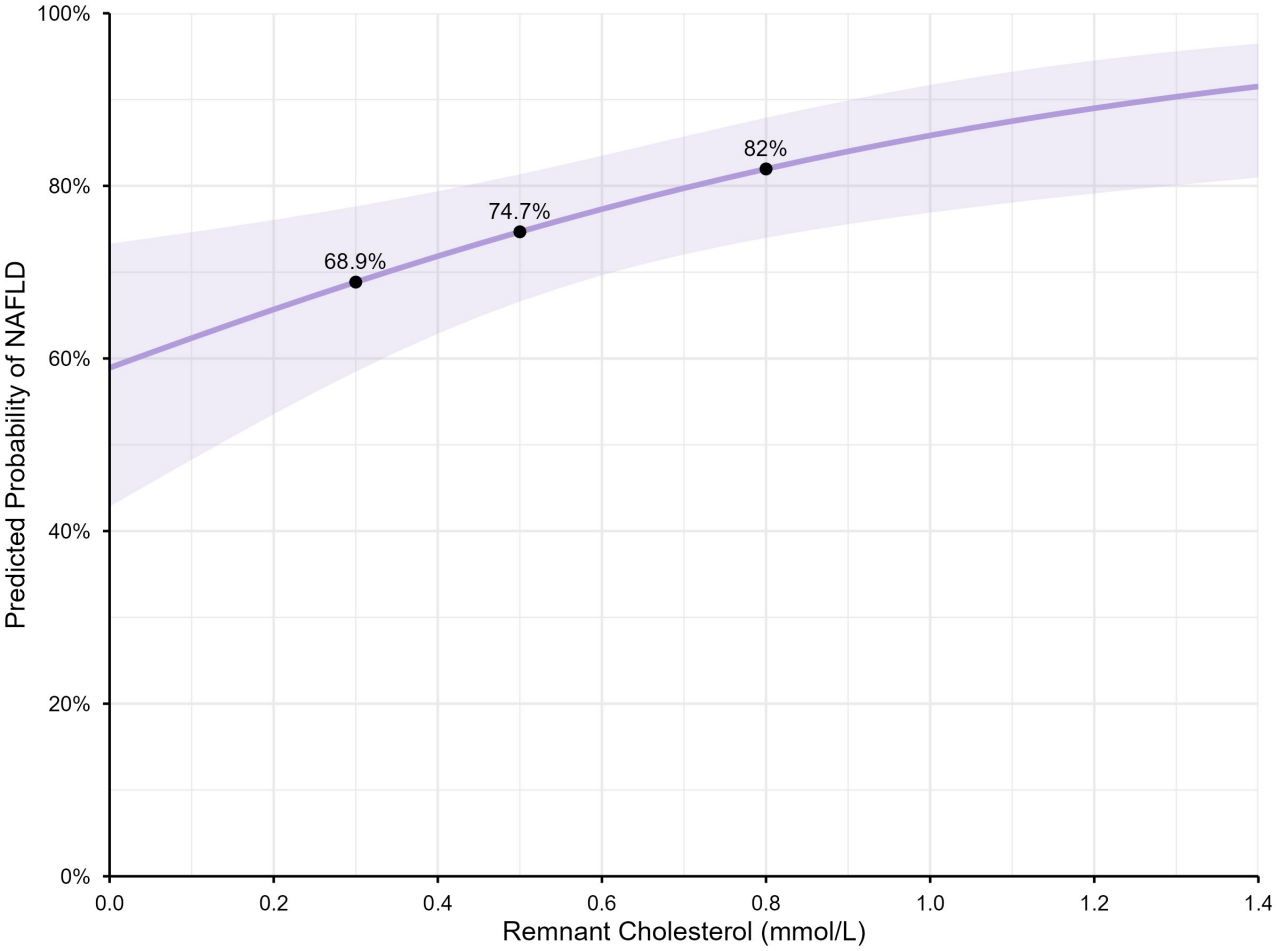
Predicted probability of RC for NAFLD. NAFLD, non-alcoholic fatty liver disease; RC, remnant cholesterol.

The median RC was 0.5 mmol/L (IQR: 0.3–0.8). The predicted probability of NAFLD increased from 68.9% (95% CI: 58.5%−77.6%) at RC = 0.3 mmol/L (the 25th percentile) to 74.7% (95% CI: 66.6%−81.3%) at RC = 0.5 mmol/L (50th percentile), and to 80.0% (95% CI: 74.0%−87.9%) at RC = 0.8 mmol/L (75th percentile) (Figure 3).

### 3.7 Sensitivity analysis

In the fully adjusted Model 3, compared with the lowest tertile, the ORs for NAFLD were 1.33 (95% CI: 0.70–2.51, *P* = 0.379) for Tertile 2 and 2.05 (95% CI: 1.06–4.03, *P* = 0.035) for Tertile 3, with a significant linear trend detected (*P* trend = 0.035). These results were consistent with the primary analysis using RC as a continuous variable, further confirming the robustness of our results (Table 3).

**Table 3:** Regression analysis of RC and NAFLD with RC as a tertile variable.

Models	NAFLD events/total population	OR	95% CI	*P* value	*P* for trend
Model 1		2.05	1.34, 3.18		0.001^*^
Tertile 1	60/106	Reference	–	–	
Tertile 2	72/101	1.90	1.07, 3.42	0.029	
Tertile 3	79/101	2.75	1.51, 5.13	0.001	
Model 2		1.90	1.23, 2.97		0.004^*^
Tertile 1	60/106	Reference	–	–	
Tertile 2	72/101	1.74	0.97, 5.15	0.066	
Tertile 3	79/101	2.48	1.34, 4.66	0.004	
Model 3		1.66	1.04, 2.68		0.035^*^
Tertile 1	60/106	Reference			
Tertile 2	72/101	1.33	0.70, 2.51	0.379	
Tertile 3	79/101	2.05	1.06, 4.03	0.035	

Data were expressed as OR (95% CI). ^*^P < 0.05 indicates statistical significance.

Model 1: crude model.

Model 2: adjusted for age and diabetes course.

Model 3: adjusted for age, diabetes course, BMI, UA, albumin. BMI, body mass index; CI, confidence interval; NAFLD, non-alcoholic fatty liver disease; OR, odds ratio; RC, remnant cholesterol; UA, uric acid.

## 4 Discussion

Disorders of lipid metabolism are recognized as significant risk factors for both macrovascular and microvascular complications associated with diabetes, as well as for the development of fatty liver disease. RC serves as a non-traditional lipid indicator that can be easily calculated and assessed. Previous research has explored the association between RC and various metabolic diseases, including NAFLD. In a study involving 6,634 participants with an average follow-up of 43.34 months, Cheng et al. (9) found that TG and RC—rather than TC or LDL-C—were independent risk factors for NAFLD. This association was particularly prominent in middle-aged and elderly subgroups, specifically among female patients with a moderate BMI and no history of cardiovascular disease or diabetes (9). Additionally, a study from an Italian hospital involving 798 patients with cardiovascular metabolic diseases reported a NAFLD prevalence of 79.2% among participants, suggesting that there was a correlation between RC and the severity of liver disease (10). Similarly, adolescents with elevated RC levels showed more pronounced hepatic fat accumulation compared with those with lower RC levels (11).

Numerous studies have investigated the relationship between RC and T2DM. In patients with T2DM, elevated RC levels are associated with an increased risk of diabetic retinopathy and peripheral arterial disease. Because of its potent atherogenic properties (12–14), high RC levels significantly heighten the risk of cardiovascular diseases. Research has demonstrated that RC concentrations are markedly elevated in patients with newly diagnosed T2DM and are closely correlated with the degree of insulin resistance. For individuals aged 18–40, an RC level exceeding 0.32 mmol/L is linked to a significantly increased odds of developing T2DM (15). However, Chen et al. reported that RC displays a non-linear association with NAFLD (16).

Regardless of whether RC was treated as a continuous or a tertile variable, it showed a positive correlation with NAFLD. This relationship is characterized by a complex interplay involving lipid metabolism, inflammatory responses, and insulin resistance, among other contributing factors. First, a reduction in lipoprotein lipase activity results in the inadequate clearance of triglyceride-rich lipoproteins, leading to lipid accumulation within hepatocytes (17, 18). Secondly, RC may provoke liver inflammation, fibrosis, and oxidative stress, thereby exacerbating the pathological progression of NAFLD (19, 20). Finally, elevated RC levels are correlated with insulin resistance, which is a critical determinant in the onset and progression of NAFLD (21, 22).

In the subgroup analyses, we observed that RC was significantly associated with NAFLD in participants aged < 60 years, males, those with a diabetes duration ≥10 years, and participants without obesity. In contrast, no correlation was found in participants aged ≥60 years, females, those with a diabetes duration < 10 years, or those with obesity. However, the interaction tests revealed no statistically significant differences for any subgroup (all *P* interaction >0.05), indicating that the effect of RC on NAFLD remained consistent across these populations. The lack of statistical significance in some subgroups, such as females, may be attributable to relatively small sample sizes and limited statistical power rather than an absence of association. Several mechanisms may explain the observed patterns within specific subgroups; for instance, in adults aged ≥60 years, multiple comorbidities may exist. These factors could potentially influence the relationship between RC and NAFLD. In younger patients, the cumulative burden of metabolic damage (including fatty liver) is generally lower; accordingly, a strong risk factor, such as elevated RC, can demonstrate a more pronounced association with the disease. Regarding sex differences, although the interaction was not significant, the point estimate was higher in males than in females. This trend might be partially attributable to the protective role of estrogen in women, which enhances insulin sensitivity, modulates lipid metabolism, and potentially inhibits hepatic steatosis. Additionally, men are more prone to central obesity and visceral fat accumulation, both of which lead to insulin resistance and promote the development of NAFLD. For patients with diabetes, a longer disease duration often correlates with declining pancreatic β-cell function and less optimal glycemic control. Long-term glucotoxicity and lipotoxicity can cause cumulative liver damage. In this context, the harmful effects of elevated RC are compounded by the metabolic damage caused by prolonged hyperglycemia, resulting in more pronounced pathogenicity. Conversely, if the disease duration is relatively short, β-cell function may still be preserved, and the pathogenic effect of RC might be masked by better glycemic control or other interventions. The differences in the relationship between RC and NAFLD across BMI subgroups are also noteworthy. Among non-obese patients with T2DM, RC—a core indicator of dyslipidemia—may serve as a key driver for the development of NAFLD. This suggests that even in the absence of obesity, hyperlipidemia remains a significant threat to liver health. In patients with obesity, however, the presence of multiple existing metabolic impairments or a potentially insufficient sample size may have obscured the specific effect of RC.

This study had several strengths. First, we simultaneously included two prevalent metabolic disorders (T2DM and NAFLD), drawing attention to this critical comorbidity model. Second, in addition to using regression models to explore the relationship between RC and NAFLD, we also applied RCS to investigate the potential for a non-linear relationship. Third, sensitivity analyses were conducted to verify the robustness of the findings. Finally, subgroup analyses were used to further explore heterogeneity among different population subgroups. Despite these strengths, several limitations should be considered. First, the sample size was relatively small; a larger cohort would enhance the reliability of the statistical results. Second, in this study, NAFLD was diagnosed *via* ultrasound. Although this method is widely used and has been validated for the detection of hepatic steatosis, it cannot differentiate simple steatosis from NASH or accurately assess the stage of liver fibrosis. Consequently, we were unable to examine the association between RC and the severity of NAFLD. Future studies using liver biopsy or advanced imaging techniques, such as MRI-PDFF or transient elastography, are needed to further explore this relationship. Third, although we adjusted for several confounders (age, BMI, diabetes course, UA, and albumin) based on statistical results, residual confounding factors remain. Variables such as dietary habits, physical activity, waist circumference, insulin resistance markers (e.g., HOMA-IR), inflammatory markers, and medication use (e.g., lipid-lowering agents and antidiabetic drugs) may influence both lipid metabolism and the development of NAFLD. Future studies should incorporate these variables to further validate the association between RC and NAFLD. Lastly, as this investigation was cross-sectional, a prospective cohort study with a follow-up period would provide more robust evidence of the relationship identified in this work.

## 5 Conclusion

In conclusion, we found that RC is independently associated with NAFLD in patients with T2DM. As an easily accessible parameter derived from standard lipid profiles, RC may serve as a simple and cost-effective marker for NAFLD risk stratification in this population. However, these findings do not indicate causality. Future prospective cohort studies are needed to determine whether the measurement of RC can improve the predictive ability for NAFLD risk beyond traditional risk factors. Randomized controlled trials should be designed to verify whether lowering RC levels can effectively reduce the incidence of NAFLD. In addition, the potential biological mechanisms involved require further investigation.

## Data Availability

The raw data supporting the conclusions of this article will be made available by the authors, without undue reservation.
