# Supplementary material for: Elevated remnant cholesterol is linked to non-alcoholic fatty liver disease in patients with type 2 diabetes mellitus
Source: Front Nutr. 2026 Apr 16;13:1782646. doi: 10.3389/fnut.2026.1782646 (PMC13128608; doi:10.3389/fnut.2026.1782646)
Supplement: Supplementary file 1 [file Table_1.docx]

**Supplementary material 1 Multicollinearity diagnosis of variables included in the regression analysis.**

| **Variable** | **GVIF** |
| --- | --- |
| Age | 1.20 |
| BMI | 1.04 |
| Diabetes course | 1.10 |
| RC | 1.02 |
| UA | 1.04 |
| Albumin | 1.08 |

**Note:** GVIF: generalized variance inflation factor.
